# The Association of Helicobacter pylori in the Oral Cavity With Dental Caries in Patients With and Without Gastric Infection: A Systematic Review

**DOI:** 10.7759/cureus.38398

**Published:** 2023-05-01

**Authors:** Nishath Sayed Abdul, Aljawharah Khalid Alkhelaiwi, Asma Awadh Alenazi, Rawan Fehaid Alrashidi, Ra’ed Ghaleb Salma

**Affiliations:** 1 Oral and Maxillofacial Surgery and Diagnostic Sciences, College of Dentistry Riyadh Elm University, Riyadh, SAU; 2 Department of Dentistry, College of Dentistry Riyadh Elm University, Riyadh, SAU

**Keywords:** infection, gastric disease, gastric infection, dental caries, helicobacter pylori

## Abstract

*Helicobacter pylori* (*H*. *pylori*) organisms are well-recognized pathogens responsible for many GI diseases. *Streptococcus mutans-*related caries and *H. pylori* infection share similar risk factors such as early childhood occurrence and low socioeconomic status. Therefore, it is possible for these two bacterial diseases to co-exist in the same environment. The present review evaluates the association of *H*. *pylori* with dental caries in patients with and without gastric infection, with the objective of comparing the association of *H*. *pylori* with dental caries in patients with and without gastric infection. A computerized literature search was performed in online databases from September 2000 to September 2022 using both electronic and manual searches for scientific databases. The research question was framed following the patient/population, intervention, comparison, and outcomes (PICO) statement. A thorough literature search identified a total of 200 manuscripts. Out of which, 100 were duplicate records and 100 were screened for eligibility, and about 78 articles were excluded, as they were not following PICO and the eligibility criterion. The retrieved 22 articles were sought for retrieval, only 17 were retrieved, and two studies did not fulfill the requirement. A total of 15 studies were recorded as eligible for the present review. There is a close association between the presence of infection of *H. pylori* in the oral cavity and the increased number of dental caries incidence in patients, even without a gastric infection. This suggests that the oral cavity is another niche for *H. Pylori* and may be the source of infection, re-infection, and transmission into the stomach.

## Introduction and background

*Helicobacter pylori* (*H. pylori*) is a spiral-shaped pathogenic bacterium found on the human gastric mucosa. It was first isolated from the gastric mucosa of a patient with gastritis for the first time in 1983 by Australian researchers Warren and Marshall. Initially, this bacterium was classified as *Campylobacter pylori,* but in 1989, it was included in a new genus, Helicobacter, and was renamed “*Helicobacter pylori*” [1]. Approximately 50% of the world’s population is infected with *H. pylori*, and the infection is significantly more prevalent in developing countries than in developed countries [2]. Epidemiological studies have demonstrated that patients with dental caries or poor oral hygiene harbor *H. pylori* in the oral cavity or gastric mucosa [3].

In the previous studies, it was reported that severe dental caries were more frequent among patients suﬀering from *H. pylori* contamination in their dental pulp. There was not much variance among the participants with chronic periodontitis and other diseases; these observations suggested that due to the colonization of *H. pylori* in the dental pulp, the microorganism is transmitted from the cavities of the carious tooth to the root canals. Hence, these carious teeth serve as an apt reservoir for *H. pylori* [4,5]. They tried to establish the relationship of these aspects with oral microﬂora and caries-producing bacterial titer. The study revealed that *H. pylori* DNA was seen in the oral cavity of 46% of the patients who showed positive results for the urease test which indicates the presence of *H. pylori* at the gastric level [6]. Similar results showed that 43% of the total 100 patients suﬀering from *H. pylori* infections in the GI tract exhibited colonization in the oral cavity [7]. Hence these authors established a relationship between dental caries, poor hygiene, and *H. pylori*.

One of the review studies conducted in Saudi Arabia stated that there was a strong association between *H. pylori* and dental caries with an OR of 1.13. Their results revealed that *H. pylori* could be the main cause of periodontal diseases [8]. Its unique spiral shape and motility allow it to move through the acidic environment of the stomach, and it has a cell wall composed of peptidoglycan and lipopolysaccharide molecules that protect it from the harsh environment [9]. *H. pylori* produces enzymes such as urease that help neutralize stomach acids and virulence factors like VacA and CagA that allow it to evade the host immune system and damage the gastric epithelium [10]. Its spiral shape allows it to move through the mucus layer covering the gastric epithelium, and its rotating flagellar motor facilitates this movement. *H. pylori* is a microaerophile that requires low levels of oxygen to survive and produces catalase to break down reactive oxygen species. It has a diameter of approximately 0.5 micrometers and a length of 2-5 micrometers, and it can form biofilms on the surface of the gastric epithelium [11].

There is evidence that suggests that *H. pylori* can be present in the oral cavity, although the nature of this association is not yet fully understood [12]. Studies have shown that *H. pylori* DNA can be detected in oral samples, such as dental plaque, saliva, and tonsillar tissue. It is believed that the bacterium may be transmitted from the stomach to the oral cavity through vomiting or regurgitation, or it may be present in the oral cavity before colonization of the stomach [11,12]. Some studies have suggested that *H. pylori* in the oral cavity may be associated with an increased risk of stomach infection, while others have not found a significant association. However, more research is needed to fully understand the relationship between *H. pylori* in the oral cavity and the development of stomach infections [12]. Additionally, the presence of *H. pylori* in the oral cavity may have implications for dental health and the potential transmission of the bacterium to other individuals through close contact [13].

The transmission of *H. pylori* from one human to another via the gastro-oral, oral-fecal, and oral-oral routes is generally acknowledged. The oral cavity is the first entry point for *H. pylori* into the human body, and researchers are very concerned about how the oral cavity contributes to *H. pylori* infection of the human body. Additionally, according to some studies, *H. pylori* in the oral cavity may have a negative impact on the way eradication therapy works clinically [13], and oral *H. pylori* is thought to be a risk factor for the recurrence of gastric *H. pylori* infection [14]. As a result, it is speculated that *H. pylori* gastric reinfection could originate from the oral cavity [14].

Moreover, *H. pylori* may also be present in the dental biofilm, which is a complex community of microorganisms that form on the surface of teeth [15]. Some studies have suggested that *H. pylori* may be more likely to colonize the dental biofilm in individuals with poor oral hygiene and that the bacterium may be more resistant to certain antimicrobial agents when present in the biofilm [16]. This may have implications for the treatment of *H. pylori* infections and the development of antibiotic resistance. While the association between *H. pylori* and the oral cavity is not fully understood, there is evidence that suggests that the bacterium may be present in oral samples and may be associated with the development of periodontal disease. Further research is needed to fully understand the implications of *H. pylori* in the oral cavity for both oral and systemic health. Therefore, this systematic review targeted this noticeable literature gap and provided valuable insights into the relationship between oral and systemic health. This review was also important for clarifying the role of *H. pylori* in dental caries, identifying potential risk factors for systemic infection, and guiding future research in this area. The purpose of the review was to evaluate the association between *H. pylori* in the oral cavity and the occurrence of dental caries in patients with and without gastric infection.

## Review

Study design

**Figure 1:**
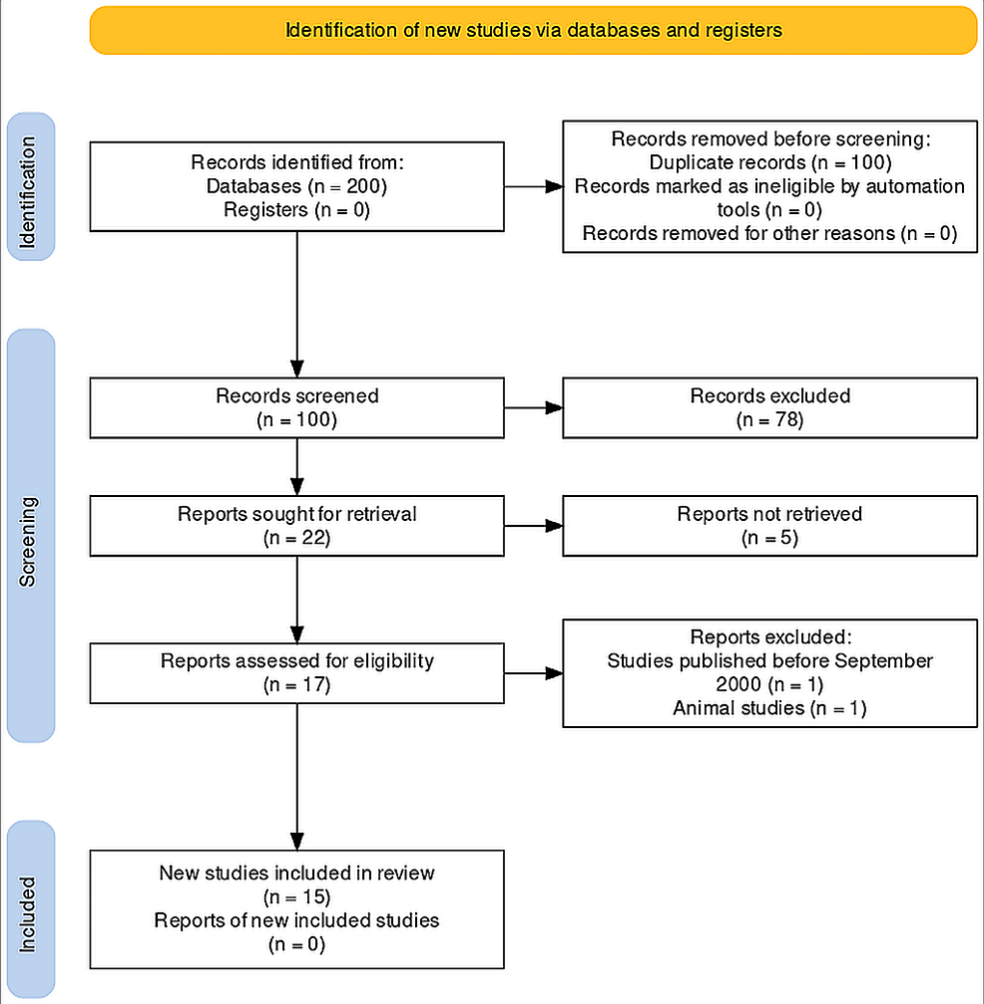
PRISMA protocol for the study

This present systematic review was carried out with a literature search that included original full-text articles and cross-sectional, case-control, observational, and descriptive studies published between September 2000 and September 2022, which evaluated the association of *H. pylori* and dental caries in patients with and without gastric infection. The study was registered with the review board of the research center of Riyadh Elm University and obtained an IRB approval number “FRP/2022/471/832/790” and PROSPERO registration number CRD42023415215 and conducted as per the PRISMA guidelines [17] (Figure 1).

Strategy for database search

In order to conduct a comprehensive search, several databases including MEDLINE, EMBASE, PubMed, Scopus, and the Cochrane Library were searched using various combinations of MeSH keywords and Boolean operators. The search strategy included the keywords "Helicobacter pylori" or "H. pylori" in combination with "Mouth," "Oral Hygiene," "Dental Plaque," and "Dental Caries" to identify articles reporting the association between *H. pylori* in the oral cavity and dental caries in patients with and without gastric infection. For MEDLINE, EMBASE, PubMed, and the Cochrane Library, the search was further narrowed by adding the keywords "Stomach Diseases," "Gastric Mucosa," and "Gastritis" to identify articles specifically reporting on patients with gastric infection. The same approach was applied to Scopus, with the addition of the "TITLE-ABS-KEY" tag to ensure that the search terms appeared in the title, abstract, or keywords of the article. Articles published before September 2000, those published in languages other than English, review studies, animal studies, and studies not adhering to the patient/population, intervention, comparison, and outcomes (PICO) framework were excluded from the search.

Selection criteria

PICO Search Strategy

The population of interest for this systematic review includes patients with or without *H. pylori* infection and with dental caries. The intervention of interest is the presence of *H. pylori* in the oral cavity. The comparison group will be patients with *H. pylori* infection compared to those without *H. pylori* infection. The primary outcome of interest is the association between *H. pylori* in the oral cavity and dental caries in patients with and without gastric infection. By examining the available literature on this topic across multiple databases, this systematic review aims to provide a comprehensive understanding of the relationship between *H. pylori* in the oral cavity and dental caries, which can have important implications for clinical management and prevention strategies for both conditions.

Inclusion Criteria

For this systematic review, studies published between September 2000 and September 2022 were included. The study participants could be of any age, gender, or ethnicity. Studies that adhered to the PICO framework were included for analysis. The review aimed to examine the relationship between dental caries and *H. pylori* in patients with and without gastric infection. Therefore, full-text articles including case-control, cross-sectional, and observational studies of the relationship between *H. pylori* and dental caries in patients with and without gastric infection were included in the review. By including studies that met these criteria, the systematic review aimed to provide a comprehensive understanding of the current literature on the association between *H. pylori* in the oral cavity and dental caries in patients with and without gastric infection, studies published in English, were approved by ethics committees, and provide relevant data. The selection of articles was done as per the STROBE criterion [18].

Exclusion Criteria

For this systematic review, several exclusion criteria were applied to ensure that only relevant studies were included for analysis. Studies published before September 2000 were excluded from this review. The review did not include articles that consisted of only titles, conference abstracts, editorial papers, review studies, and animal studies. Only studies that adhered to the PICO framework were included in this review. Furthermore, studies that were not related to *H. pylori*, dental caries, dental plaque, oral hygiene, and gastric infection were also excluded from the review. By applying these exclusion criteria, the systematic review aimed to ensure that only studies that were relevant to the research question were included for analysis, providing a comprehensive understanding of the relationship between *H. pylori* in the oral cavity and dental caries in patients with and without gastric infection.

Collection and Data Extraction

The initial search strategy was divided into two stages: In the first stage, the authors were independently involved in the process and extracted the necessary data. All the available titles and abstracts were identified, scanned, and included in those studies that fulfilled the inclusion criterion. In the second stage, full-text articles were thoroughly investigated by two reviewers independently, and conflicts arising between the two were resolved by a third reviewer. The disagreements were discussed among the remaining authors, and the consensus was made before the inclusion of the studies in this review.

Control of Bias Assessment

The following issues will be included in the risk of bias or quality assessment in the present systematic review: (1) Completeness of article information on *H. pylori* (2), selective outcome reporting (3), outcome measures (association of *H. pylori* with dental caries with and without gastric infection), (4) study design, and (5) conflict of interest in the conduct of the study. The authors screened the titles and abstracts based on eligibility criteria. Full-text articles of the selected were then obtained and evaluated. The characteristics of each article, such as the location of the study, examination site, technique, evaluator, and findings, were examined.

**Figure 2:**
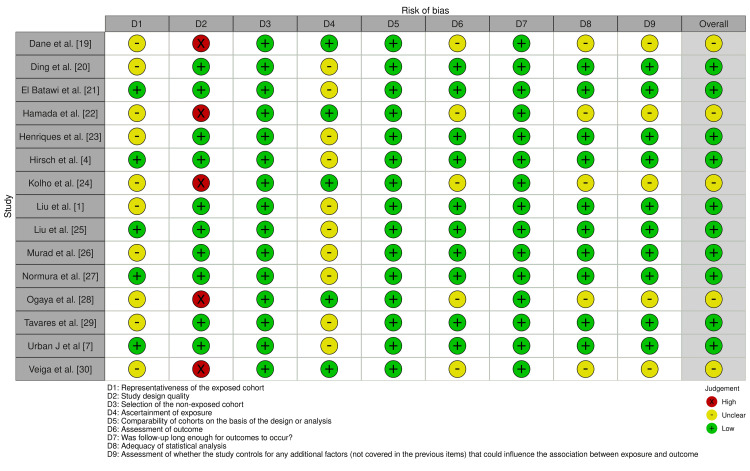
Risk of bias assessment for all the articles in the study

For the methodological quality evaluation of the included studies, Newcastle-Ottawa Scale criteria were employed to determine the quality of the articles (Figure 2).

Each study received a grade ranging from 0 to 9. Studies with nine to seven stars were deemed to be of high quality, studies with four to six stars to be of moderate quality, and studies with one to three stars to be of low quality and highly susceptible to bias.

Data Synthesis

Text and tables summarize and explain the characteristics of the findings in the included studies. The following variables were extracted, and validation checks were performed by two reviewers to assess the accuracy of the extracted fields: (1) first author and year of publication, (2) target condition, (3) study design and sample size, (4) country and setting of the study, (5) technologies/diagnostic method used, and (6) findings/result. The present review could not be converted to a meta-analysis as the included articles did not provide the required quantitative data for analysis, and due to the lack of homogeneity of the available data, a systematic review of the data was performed.

A thorough literature search identified a total of 200 manuscripts. Out of which, 100 were duplicate records and 100 were screened for eligibility. About 78 articles were excluded as they were not following PICO and the eligibility criterion. The retrieved 22 articles were sought for retrieval, and only 17 were retrieved, and two studies did not fulfill the requirement. A total of 15 studies were recorded as eligible for the present review. Table 1 presents the literature search process as per PRISMA guidelines.

**Table 1:** Various studies establishing the correlation between H. pylori and dental caries PCR: polymerase chain reaction

S.no.	Author	Year	Country	Sample size	Average age (in years)	Samples in the oral cavity	With gastric infection	Correlation between *H. pylori* and dental caries	Detection method used for *H. Pylori* and study type
1	Dane et al. [19]	2016	Turkey	35	5 to 15		Not mentioned	No signiﬁcant diﬀerences obtained	Endoscopy
2	Ding et al. [20]	2015	China	1050			Without gastric infection	Positive	*H. Pylori* antigen test
3	El Batawi et al. [5]	2020	UAE	48	4 to 7	Dental cavities	Without gastric infection	Positive	PCR cross-sectional study
4	Henriques et al. [21]	2016	Brazil	40			Without gastric infection	Positive	Checkerboard DNA-DNA
5	Hamada et al. [22]	2019	Japan	87			Not mentioned	Positive	PCR
6	Hirsch et al. [4]	2012	Germany	10		Oral cavity	Not mentioned	Dental caries positive	PCR cross-sectional study
7	Kolho et al. [23]	2001	Finland	56	4.5 to 16	Oral cavity	Not mentioned	Dental caries positive	GI endoscopy, case-control study
8	Liu et al. [1]	2008	China	214	3-6 years	Dental plaque	Without gastric infection	Dental caries positive	PCR
9	Liu et al. [24]	2013	China	841	18 to 74	Dental plaque	Not mentioned	Dental caries positive	PCR
10	Murad et al. [25]	2014	Brazil	36	27 to 66		With gastric	Positive	Checkerboard DNA-DNA hybridization
11	Normura et al. [26]	2020	Japan	131			Without gastric infection	Positive	PCR
12	Ogaya et al. [27]	2015	Japan	40		Oral specimens	Without gastric infection	Positive	PCR
13	Tavares et al. [28]	2011	Brazil	40	6.8	Oral samples	With gastric infection	Negative/no caries	Checkerboard DNA-DNA hybridization
14	Urban J et al [7]	2017	Poland	108	18>	Oral samples	Gastric positive	Positive	PCR
15	Veiga et al. [29]	2015	Portugal	447	12 to 19		Without gastric infection	Negative > positive correlation	PCR

The present review could not be converted to a meta-analysis as the included articles did not provide the required quantitative data for analysis, and due to the heterogeneity of the available data, this study is only a systematic review.

Discussion

A systematic review such as this addresses a significant literature gap in our understanding of the role of *H. pylori* in oral health. The review synthesized the available evidence on the relationship between *H. pylori* in the oral cavity and dental caries, a common oral disease characterized by the demineralization of tooth structure caused by acid produced by bacteria in dental plaque. By examining studies in patients with and without gastric infection, this review also explored the potential relationship between *H. pylori* in the oral cavity and systemic infection. This is important because *H. pylori* is known to colonize the stomach and cause a range of GI disorders, and there is evidence that suggests that oral colonization may be a risk factor for gastric infection. The review was important for several reasons. First, it helped clarify the relationship between *H. pylori* in the oral cavity and dental caries, which has been the subject of conflicting research [6]. Second, it also contributed to our understanding of the potential role of *H. pylori* in systemic infection, which has important implications for public health. Moreover, several key gaps in the existing literature were identified and highlighted for future studies employing a similar protocol as ours.

Table 1 presents a summary of different studies on the correlation between *H. pylori* infection in the stomach and dental caries in the oral cavity. *H. pylori* is a bacterial species that infects the stomach lining and is a well-known cause of gastritis, peptic ulcers, and gastric cancer. The studies have been conducted in different countries such as Turkey, China, UAE, Brazil, Germany, Finland, Japan, and Portugal, with different sample sizes ranging from 10 to 1050 individuals. Most studies used PCR as the detection method for *H. pylori*, while some studies used *H. pylori* antigen test, endoscopy, or checkerboard DNA-DNA hybridization. The average age of participants in these studies varied from 3 to 74 years, with some studies focusing on children and others on adults. Among the 15 studies listed, only four studies reported a positive correlation between *H. pylori* infection and dental caries, while five studies reported no significant correlation. The remaining six studies reported mixed or inconclusive results. It is worth noting that most studies did not find any significant difference in dental caries between individuals with and without *H. pylori* infection. The studies included in Table 1 provide conflicting evidence on the correlation between *H. pylori* in the oral cavity and its association with gastric infection. Some studies have reported a positive correlation between *H. pylori* in the oral cavity and gastric infection, while others have found no significant association. Studies by Ding et al. [20], Murad et al. [25], and Urban J et al. [7] found a positive correlation between *H. pylori* in the oral cavity and gastric infection, while studies by Dane et al. [19] and Tavares et al. [28] found no significant association. The remaining studies included in the table found mixed results. The implications of these findings are significant, as they suggest that *H. pylori* transmission from the oral cavity to the stomach may not be a primary mode of infection. While some studies suggest that *H. pylori* in the oral cavity may be a potential source of infection, others suggest that it is not significant. It is essential to note that the studies included in the table varied in their sample size, detection method, and study design, which may contribute to the conflicting results.

Future studies with larger sample sizes, standardized detection methods, and well-designed study protocols are necessary to confirm the correlation between *H. pylori* in the oral cavity and gastric infection.

*H. pylori* is an S-shaped or curved gram-negative rod that is ubiquitous and is associated with various types of disorders, including coronary heart disease, thyroid disease, anemia, diabetes mellitus, dyslipidemia, gastric lymphoma, and hypothyroidism [30,31]. The primary mode of transmission of the microorganism is not distinctly elucidated, even though they could be detected in the stomach of almost every individual across the globe. The transmission mechanism could be due to iatrogenic causes, feco-oral routes, and oral-oral routes in the industrialized and developing world with the help of food and water acting as transporting agents or could also exist naturally in the environment [32,33]. These previous studies reported the association of *H. pylori* in the oral cavity with the existence of cariogenic [4,22,24].

The action of *H. pylori* culture supernatant on the dual-species bioﬁlm consisting of *S. mutans* and *S. sanguinis* was evident. They assessed its possible capacity to distress the dental hard tissue. These authors demonstrated that *H. pylori* supernatant inhibits both *S. mutans* and *S. sanguinis* bioﬁlms (*S. sanguinis* inhibition being suggestively more signiﬁcant than *S. mutans*) [34].

Several studies have investigated the possible associations between *H. pylori* and other oral diseases besides dental caries [35-37]. For instance, some studies have suggested that *H. pylori* may be involved in the pathogenesis of periodontal disease, oral lichen planus, and oral cancer [37]. Some studies have found that the presence of *H. pylori* in the oral cavity is associated with an increased risk of periodontal disease. An article discussed the potential role of a certain bacterium typically found in the oral cavity, in the development of oral cancer [38]. However, the exact mechanism by which *H. pylori* contributes to periodontal disease is not yet clear [39]. Some studies have suggested that *H. pylori* infection may be associated with oral lichen planus, although the evidence is not yet conclusive [39,40].

Infection with *H. pylori* is thought to be the primary cause of chronic gastritis and is closely associated with gastric and duodenal cancer. Due to the tight connection between the oral cavity and the digestive tract, research into the connections between oral H*, pylori* infection, dental caries, and peptic ulcer disease has focused on the interaction between oral *H. pylori* and illnesses of the oral cavity. Although *H. pylori* is known to be linked to a number of digestive tract disorders, it is unclear how these disorders relate to oral cavity disorders [35,41]. Many studies have attempted unsuccessfully to cultivate *H. pylori* from the oral cavity since Krajden and Ferguson originally isolated *H. pylori* from dental plaque and saliva [42,43]. This is likely because there are so many oral bacteria that it is difficult to isolate *H. pylori*. Due to its excellent sensitivity and specificity, PCR is now commonly employed to identify oral *H. pylori* [24].

According to one study, the prevalence of dental caries was 61.36% overall and 62.90% and 59.60% in men and women, respectively. *H. pylori* was found in 68.76% of the samples from men, 67.68% from women, and 68.25% from all samples. Dental caries can be readily caused by poor oral hygiene practices. This trend may be brought on by inflammation, age, and poor dental hygiene practices. Participants who tested positive for oral *H. pylori* had a significantly greater frequency of dental caries than participants who tested negative. In this cross-sectional investigation, dental caries and *H. pylori* infection were related [24].

Limitations

The review had several limitations that should be considered when interpreting its findings. Firstly, the study had inclusion and exclusion criteria which could have potentially led to selection bias. Only studies that were published in the English language and adhered to the PICO framework were included, and studies that were published before September 2000, were in a language other than English, or were animal studies were excluded. This could have led to the exclusion of relevant studies and may limit the generalizability of the findings. Secondly, the studies included in the review used different methods to diagnose *H. pylori* infection and dental caries. The lack of standardization in diagnostic methods could have resulted in different rates of diagnosis and potentially influenced the association between *H. pylori* and dental caries. In addition, most of the studies included in the review were observational studies, which cannot establish causality. Additionally, the studies were conducted in different populations with varying ages, ethnicities, and dental health status. The heterogeneity of the studies could have influenced the association between *H. pylori* and dental caries.

More importantly, the review did not consider the effect of confounding variables such as socioeconomic status, oral hygiene, and dietary habits. These variables could have influenced the relationship between *H. pylori* and dental caries, but their effects were not assessed in the studies included in the review. Therefore, while the review provides some evidence for an association between *H. pylori* and dental caries, the limitations of the included studies should be considered when interpreting the findings. Future studies should aim to address these limitations by using standardized diagnostic methods, controlling for confounding variables, and using longitudinal study designs to establish causality.

## Conclusions

There is a close association between the presence of infection of *H. pylori* in the oral cavity and an increased number of dental caries incidences in patients without gastric infection. This suggests that the infection of *H. pylori* in the oral cavity itself may cause an increase in dental caries incidence. This suggests that the oral cavity is another niche for *H. pylori* and may be the source of infection, re-infection, and transmission into the stomach.
